# Rectus Sheath Hematoma Secondary to Domestic Violence

**DOI:** 10.7759/cureus.17058

**Published:** 2021-08-10

**Authors:** Elgun Samadov, Arturan Ibrahimli, Samir Mamishov

**Affiliations:** 1 Department of Surgery, Leyla Medical Center, Baku, AZE; 2 Medicine, Ankara University Faculty of Medicine, Ankara, TUR; 3 Radiology, Mediland Hospital, Baku, AZE

**Keywords:** rectus sheath hematoma, domestic violence, abdominal pain, women violence, abdominal trauma

## Abstract

Rectus sheath hematoma (RSH) is an infrequent condition that occurs when epigastric arteries bleed into the rectus sheath and sometimes acts like an acute abdomen. In view of the fact that it is a rare case without specific clinic signs, misdiagnosis and use of invasive manipulations for patients are possible. A 27-year-old woman applied to our clinic with abdominal pain, weakness, and nausea. Acute abdominal pain was in the periumbilical region and physical examination revealed tenderness, rebound phenomenon, and local mass in the right periumbilical region. Rectus sheath hematoma was identified by magnetic resonance imaging. The cause in this case was abdominal trauma due to domestic violence. After the legal procedures, symptomatic treatment was prescribed to the patient. Interestingly in this case, we did not get to the diagnosis through history, we reached the history after diagnosis. Besides the treatment of the patient, maybe our team prevented further violence against a woman.

## Introduction

Rectus sheath hematoma (RSH) is an infrequent condition that occurs when epigastric arteries bleed into the rectus sheath and sometimes acts like an acute abdomen. The first case in the literature was reported by McKim [1]. Rectus sheath hematoma emerges from many reasons, such as anticoagulant therapies, severe exercise, and trauma. Especially in cultures in which domestic assault against women is common, abdominal trauma should be considered as a cause of RSH. Sheth et al. reported in their study that chronic kidney disease, extreme coughing, steroid and immunosuppressant therapies, and coagulopathies can also be specific reasons [2]. Abdominal trauma caused by violence against women is often skipped by many doctors because of the incorrect history of the patient. In this case, the significance of rectus sheath hematoma diagnosis in abdominal pain and the doctor’s role in preventing domestic violence are mentioned.

## Case presentation

**Figure 1:**
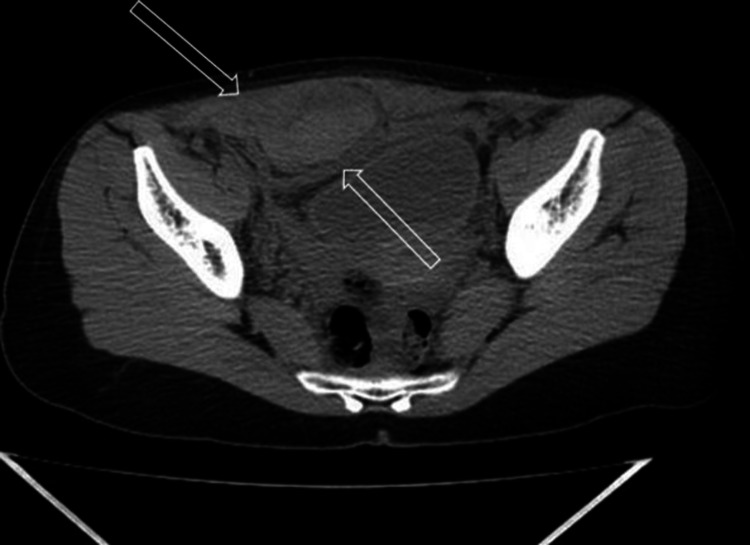
Axial native computer tomography. Right rectus abdominus muscle is markedly enlarged compared to the normal left due to a large heterodense (27 to 61 HU) mass, which extends to the subperitoneal space, causing bladder compression.

**Figure 2:**
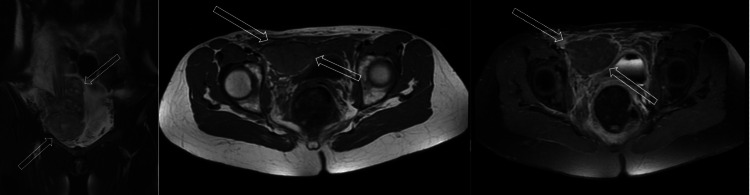
a. Coronal T2 FATSAT; b. Axial T1 sequence; c. Axial, T1 FATSAT post-contrast (gadolinium) sequence, hematoma with mixed signal changing, no contrast

A 27-year-old woman applied to our clinic with abdominal pain, weakness, and nausea. Acute abdominal pain was in the periumbilical region and physical examination revealed tenderness, rebound phenomenon, and local mass in the right periumbilical region with a 130mm x 44mm size. No sign of contusion, scar, or other skin lesions was seen in the abdominal wall. Her vital signs were heart rate 98 per minute, blood pressure 95/70 mmHg, oxygen saturation 97%, respiratory rate 12 per minute, and temperature 36.9 Celsius. Hemoglobin levels and leukocytes were 9.2 gr/dl and 13.400 IU, respectively. Initially, the patient underwent abdominal ultrasonography, which confirmed a large complex, avascular, heteroechoic mass along the lower rectus sheath. Given the described ultrasound features and in the context of this clinical presentation together with pain and palpable mass, it was suspected as a hematoma. However, because of its size, it could not be concluded with certainty whether the patient had an additional intra-abdominal pathology as a free fluid. Thus, on suspicion, an abdominopelvic computer tomography without intravenous or oral contrast was ordered. Computer tomography showed the right rectus abdominis muscle was markedly enlarged compared to the normal left due to a large heterodense (27 to 61 Hounsfield unit) mass that extended to the subperitoneal and inferiorly prevesical spaces, causing bladder compression (Figure 1). Rectus sheath hematoma was identified. After explaining to the patient about her condition, the clinical doctor was informed. Not knowing about predisposing factors or causes, to exclude undergoing neoplasia, the patient underwent pelvic magnetic resonance imaging (MRI) with an intravenous contrast agent. The rectus sheath haematoma was confirmed (as T1 and T2 mixed high signal changing), and an additional intra-abdominal pathology was excluded (Figure 2).

Initially, the patient's relatives interrupted our procedure with the patient many times and according to her first history, there was no systemic disease condition, anticoagulant or other drug admission, or trauma exposure. Thus, our team was suspicious of a tumor. However, after magnetic resonance imaging and computer tomography findings revealed rectus sheath hematoma, we could talk with the patient privately and we understood that the patient faced violence in the family several days before the symptoms occurred. The local police department was informed about the domestic violence and besides treating our patient, perhaps our team intercepted future domestic violence against the patient.

Differential diagnosis

Rectus sheath hematoma can mimic a variety of intra-abdominal pathologies, including acute appendicitis, cholecystitis, biliary colic, diverticulitis, strangulated hernia, intestinal obstruction, ovarian torsion, and abdominal aneurysm. Consequently, the importance of differential diagnosis of rectus sheath hematoma is very crucial for future approach and treatment of the patient.

Treatment

Treatment options for rectus sheath hematoma are mainly conservative and symptomatic. There are not any specific treatment options for rectus sheath hematoma. Treating underlying reasons is sufficient for symptomatic patients. In some cases, if a patient is in deep pain and is not stable hemodynamically, intraperitoneal rupture developed, or hematoma continues to enlarge, surgery should be considered [3]. A safe and less invasive option than surgery is embolisation of the artery [4]. In the literature there is a lack of prospective randomised studies about mortality rates; barely several publications show mortality rates in patients is 2% [5]. Symptomatic treatment was prescribed to our patient including non-steroid anti-inflammatory analgesics.

Outcome and follow up

At two weeks’ control check, improvement in symptoms was examined and the hematoma had contracted according to ultrasound imaging results.

## Discussion

Blood vessels of the rectus muscle originate from superior epigastric and inferior epigastric arteries. Therefore, rectus sheath hematomas occur when those arteries or their perforating branches are ruptured or damaged [6].

The most common symptom of rectus sheath hematoma is acute twinge abdominal pain with increasing intensity, frequently starting in the lower abdominal quadrant. Other relevant symptoms can be abdominal distension, hypotension, low hemoglobin levels, periumblical ecchymosis (Cullen sign), peritoneal irritation, a positive Carnett sign, vomiting, nausea, constipation, scrotal swelling, and tachycardia [7,8]. Occasionally, acute appendicitis, cholecystitis, biliary colic, diverticulitis, strangulated hernia, intestinal obstruction, ovarian torsion, and abdominal aneurysm can imitate rectus sheath hematoma [9].

Diagnosis of rectus sheath hematoma is done by imaging methods. Ultrasonography is a simple and cost-effective method to diagnose rectus sheath hematoma; the main demonstrator sign is heterogeneity. However, in some cases like obese or pregnant patients, ultrasonography is suboptimal for diagnosing and other imaging methods such as computer tomography and magnetic resonance imaging can be used to evaluate the patient. In computer tomography imaging with intravenous contrast, hematoma is evaluated by its confinement to the abdominal wall, lack of enhancement, and high attenuation. Berna et al.'s research illustrates that the role of computer tomography imaging in rectus sheath hematoma diagnosis is crucial, as sensitivity and specificity are 100% [10]. Magnetic resonance imaging with intravenous contrast is preferable when there is a suspicion for muscle tumor.

The causes of rectus sheath hematoma are variable, starting from anticoagulant usage to trauma. Sheth et al. reported in their study that chronic kidney disease, cancer, liver diseases, and abdominal injections are among the causes [2].

Abdominal traumas are also one of the main causes of rectus sheath hematoma. In our patient, the reason was abdominal trauma related to domestic violence. Since about 36% of the women who face domestic violence tend to not disclose because of ignominy, discomfiture, and disgrace, doctors ought to be more cautious when they confront a woman with abdominal trauma [11]. Various articles have been published about the increase in violence against women during the COVID-19 pandemic. Health professionals have a great responsibility in this issue; we should understand the problem, dangers, and outcomes of violence against women and it is critical to provide relevant medical services, treatment, and comprehensive care for those patients [12].

## Conclusions

Rectus sheath hematoma is a rare outcome of abdominal traumas. Thus, it is possible to misdiagnose the disease and patients can face redundant procedures. The underlying reason should be investigated thoroughly. The underlying cause in our case was domestic violence. In the publications there is an absence of studies on the percentage of abdominal traumas caused by violence against women.

Diagnostic evaluation and treatment approach in abdominal trauma-caused rectus sheath hematoma are crucial during the course of the disease.

## Appendices

Patient's Perspective

Initially, I felt a swelling and pain near my belly and felt a little discomfort. I waited for 2-3 hours so that maybe the pain will be gone but it did not and I talked with my family. We decided to go to the hospital. I entered the doctor’s room and he asked me some questions and examined me. Then he sent me to the radiologist in the hospital and told me it is necessary to have further examinations to evaluate my condition. I really got suspicious about other bad diseases such as cancer because I have read some different diagnoses on the internet according to my symptoms. After several procedures I went to the first doctor’s room again to show my diagnostic results, but I was alone when I entered the room for the second time. After he saw my results he asked me about if I had a trauma in several days. First, I felt very embarrassed to disclose the reason for my trauma. But the doctor warmingly convinced me to tell the reason. I told him that I have faced with a violence at home and had a hit to my abdomen. He asked me if I need psychological support and asked me to get into contact with social services. Then he prescribed me some medicine for my pain and told me nothing to worry about my disease.
